# wgd v2: a suite of tools to uncover and date ancient polyploidy and whole-genome duplication

**DOI:** 10.1093/bioinformatics/btae272

**Published:** 2024-04-17

**Authors:** Hengchi Chen, Arthur Zwaenepoel, Yves Van de Peer

**Affiliations:** Department of Plant Biotechnology and Bioinformatics, Ghent University, Ghent 9052, Belgium; VIB Center for Plant Systems Biology, VIB, Ghent 9052, Belgium; UMR 8198, Evo-Eco-Paleo, University of Lille, CNRS, Lille, F-59000, France; Department of Plant Biotechnology and Bioinformatics, Ghent University, Ghent 9052, Belgium; VIB Center for Plant Systems Biology, VIB, Ghent 9052, Belgium; Centre for Microbial Ecology and Genomics, Department of Biochemistry, Genetics and Microbiology, University of Pretoria, Pretoria 0028, South Africa; College of Horticulture, Academy for Advanced Interdisciplinary Studies, Nanjing Agricultural University, Nanjing 210095, China

## Abstract

**Motivation:**

Major improvements in sequencing technologies and genome sequence assembly have led to a huge increase in the number of available genome sequences. In turn, these genome sequences form an invaluable source for evolutionary, ecological, and comparative studies. One kind of analysis that has become routine is the search for traces of ancient polyploidy, particularly for plant genomes, where whole-genome duplication (WGD) is rampant.

**Results:**

Here, we present a major update of a previously developed tool wgd, namely wgd v2, to look for remnants of ancient polyploidy, or WGD. We implemented novel and improved previously developed tools to (a) construct *K*_S_ age distributions for the whole-paranome (collection of all duplicated genes in a genome), (b) unravel intragenomic and intergenomic collinearity resulting from WGDs, (c) fit mixture models to age distributions of gene duplicates, (d) correct substitution rate variation for phylogenetic placement of WGDs, and (e) date ancient WGDs via phylogenetic dating of WGD-retained gene duplicates. The applicability and feasibility of wgd v2 for the identification and the relative and absolute dating of ancient WGDs is demonstrated using different plant genomes.

**Availability and implementation:**

wgd v2 is open source and available at https://github.com/heche-psb/wgd.

## 1 Introduction

Ancient polyploidizations, resulting from whole-genome duplication (WGD), have been uncovered in most plant lineages, including angiosperms (Albert *et al.* 2013, Van de Peer *et al.* 2017, Yang *et al.* 2020), gymnosperms (Liu *et al.* 2022), monilophytes (Chen *et al.* 2023), lycophytes (Wickell *et al.* 2021), and bryophytes (Healey *et al.* 2023). However, the inference of ancient WGDs can be challenging. The identification and delineation of genomic collinearity, where sets of duplicated genes from homeologous genomic regions (intragenomic regions that are homologous because of WGD or hybridization) show conserved gene order, has been among the first methods to find evidence for ancient WGD (The Arabidopsis Genome Initiative 2000, Vandepoele *et al.* 2002, Van de Peer 2004). The construction of so-called age distributions of gene duplicates, which does not require structural genome information, has been another important approach for identifying putative ancient WGDs (Blanc and Wolfe 2004, Van de Peer 2004, Maere *et al.* 2005, Cui *et al.* 2006). In such age distributions, where the number of duplicates in a genome is plotted as a function of their age (measured by the number of synonymous mutations per synonymous site), WGD events will become visible as peaks against a background distribution coming from small-scale gene duplications (Lynch and Conery 2003), as such an event creates numerous duplicates at the same time (Van de Peer 2004). A last major approach to find evidence for ancient WGD events is phylogenomic gene-tree/species-tree reconciliation. In this approach, where gene duplicates are mapped onto a species tree, an overabundance of duplication events mapped to a specific branch of the species tree can be indicative for a WGD event (Jiao *et al.* 2011, Zwaenepoel and Van de Peer 2019). While many of the above approaches for the identification of WGDs allow to greater or lesser extent also the relative dating (i.e. phylogenetic placement) of ancient WGDs, a much more challenging task is the absolute dating of WGD events (Doyle and Egan 2010), which is important for revealing the geological epoch at which these WGDs occurred and to see whether polyploidization events correlate with paleo-climatic changes, mass extinction events or key innovations (Van de Peer *et al.* 2017).

Here, we present an improved and integrated pipeline, i.e. wgd v2, for the identification and dating of ancient WGDs. wgd v2 substantially improves and expands on its predecessor (Zwaenepoel and Van de Peer 2018), consisting of a suite of user-friendly tools that include improved methods to delineate the paranome, to construct various plots showing intragenomic and intergenomic collinearity, to build *K*_S_ age distributions, to correct for unequal substitution rates across lineages in the phylogenetic placement of WGDs, to date WGDs, and more. We showcase wgd v2 by an application on the recently published seagrass genome *Posidonia oceanica* (Ma *et al.* 2024) and some other plant genomes with previously well-documented ancient WGDs. We hope that our new and improved tool will prove useful in the search for remnants of ancient polyploidy and WGDs in plant and other genomes.

## 2 Materials and methods

### 2.1 Improved delineation of the paranome

In wgd v2, we correct the gene length biased bit-score in sequence similarity searches for the improvement of whole-paranome (all duplicates) inference. Based on the initial hits from the all-against-all similarity searches using diamond (v0.9.14.115) (Buchfink *et al.* 2021), the correction is the same as in OrthoFinder (Emms and Kelly 2019) but with additional customized settings: (i) first, the proxy gene length of each hit is calculated as the product of the target and query gene length in amino acids; (ii) second, a user-defined number of bins is used to split the hits, with default 100. A user-defined percentage of upper hits per bin, with default 5, can be set to control the percentile of hits for subsequent regression analysis. The associated gene length and bit-score of the upper hits per bin are then concatenated and fitted to a linear least-squares regression after log-transform. The bit-scores are then normalized as the ratio of original bit-score to the expected bit-score given the gene length and deduced linear regression function. The Markov Cluster algorithm (mcl v10-201) (Dongen 2008) is then used with the normalized bit-scores using a user-defined inflation factor (default 2.0), to group sequentially similar genes into gene families. The above three steps, including the diamond search, the bit-scores normalization and the mcl clustering, are achieved by a single command in the program, i.e. “wgd dmd.”

To compare the accuracy of updated whole-paranomes to previously inferred whole-paranomes without normalization, we further identified mis-assigned genes based on the presence of shared protein domains identified by HMMER (v3.1b2). The procedure was as follows. Firstly, MAFFT (v7.310) (Katoh and Standley 2013) was run on each gene family with the parameter set as “-auto” to acquire an amino acid multiple sequence alignment (MSA) file. From each MSA file, a hidden Markov model (HMM) profile was constructed using the “hmmbuild” program. Based on the obtained profiles, a profile database was then built using the “hmmpress” program. Amino acid sequences of each gene family were searched against the established HMM profile database using the “hmmscan” program with E-value threshold set as 1e-10. Genes which had no hit against any protein domain were identified as mis-assigned genes. The subsequent comparison of mis-assigned genes between the updated and the previously inferred gene families without normalization was performed at different percentiles of gene family sizes.

### 2.2 Improved representation of intraspecific and interspecific collinearity

To further increase the potential of uncovering genomic collinearity towards finding remnants of WGDs, three new genome collinearity visualization methods were implemented in wgd v2. First, “dupStack plots” visualize so-called multiplicons (homeologous segments) along chromosomes. Multiplicons are reconstructed from collinearity searches conducted with i-ADHoRe (v3.0.01) (Proost *et al.* 2011) with user-defined minimum length of considered scaffolds and segments. Second, “Syndepth plots” extract segment information to show intraspecific and interspecific collinearity ratios per species pair. Third, a gene homology matrix dot plot (Sonnhammer and Durbin 1995) visualization was implemented to show the distribution of homologous and homeologous gene pairs throughout the genome. The three methods are automatically called in the program “wgd syn” and can be used by means of “wgd viz.”

### 2.3 Construction of *K*_S_ age distributions

Given the full paranome, the construction of so-called *K*_S_ age distributions (Maere *et al.* 2005, Chen and Zwaenepoel 2023) is achieved in three steps by the “wgd ksd” program. First, an amino acid MSA is built for every gene family using a user-defined alignment program (default mafft and “–auto”), which is then back-translated into a codon-level nucleotide MSA. Second, for each gene family, a maximum likelihood estimate of *K*_S_ for each pair of genes is obtained using the CODEML program from the PAML (v4.9j) package with preset parameters following (Vanneste *et al.* 2012). Finally, for every gene family, a phylogenetic tree is constructed using either FastTree (Price *et al.* 2010) or IQTREE (Nguyen *et al.* 2014) (set by the option “tree_method”) and then rooted using midpoint rooting, or by average linkage clustering. The subsequent step to remove redundancy is achieved in either node-weighted or node-averaged manner by the option “—node_average,” such that the weights of a single gene duplication event sum up to 1, or a single gene duplication event is represented by a single averaged *K*_S_ value (Chen and Zwaenepoel 2023). The anchor pair (pairs of duplicates resulting from a WGD event and residing in duplicated, i.e. homeologous, segments) *K*_S_ distribution can be further obtained using “wgd syn” given the genomic coordinates of the protein-coding genes in General Feature Format (GFF).

The construction of *K*_S_ age distributions for orthologous genes, reflecting speciation events, rather than duplication events (see further), can be achieved in the same way using orthologous, rather than paralogous, gene families.

### 2.4 Correcting for substitution rate variation

The phylogenetic location of putative WGD events can be inferred by comparing the order of speciation events and WGD events, which are represented as peaks in the *K*_S_ distributions for orthologous and paralogous genes, respectively. However, such a comparison may lead to biased results when variation in substitution rates across evolutionary lineages is not considered (Smith and Donoghue 2008, Sensalari *et al.* 2021). In wgd v2, the correction of different substitution rates for different species was implemented as described before (Sensalari *et al.* 2021) but we modified the calculation of the standard deviation (std) of the corrected age to take into account the covariance within and across sets of three species (so-called “trios”). The procedure is as follows: given a species tree, first, “trios of species” are considered, composed of the “focal” species, a sister species, and an outgroup species. Second, for all pairwise comparisons within a trio, we obtain 200 bootstrap replicates for the orthologous *K*_S_ distribution to estimate the mean and standard deviation of the mode (the *K*_S_ value at which the estimated density reaches the maximum) of the kernel density estimate (KDE). The adjusted divergence time between a specific focal and sister species pair will be represented by the averaged rescaled mode of all possible trios (i.e. all the possible outgroups given the focal and sister species pair) calculated using,

(1) $$\mathrm{rescaled}\;\mathrm{mode}=\sum_{i=1}^{N}\frac{\begin{array}{c} \mathrm{Mean}\left(\mathrm{focal},\mathrm{outgroup}\left(i\right)\right)\\ -\;\mathrm{Mean}\left(\mathrm{sister},\mathrm{outgroup}\left(i\right)\right)\\ +\;\mathrm{Mean}\left(\mathrm{focal},\mathrm{sister}\right) \end{array}}{N}$$

where *N* is the number of all possible trios. The Mean (*i*, *j*) represents the averaged mode of KDE fitted on the 200 bootstrap replicates of the orthologous *K*_S_ distribution between species *i* and *j* for each trio. To represent the uncertainties associated with the adjusted divergence time, its standard deviation is calculated as Equation (2):

(2) $$\mathrm{std}=\frac{1}{N}\sqrt{\sum_{i=1}^{N}\mathrm{Var}\left(\mathrm{trio}\left(i\right)\right)+2\sum_{i=1}^{N-1}\sum_{j=i+1}^{N}\mathrm{Cov}\left(\mathrm{trio}\left(i\right),\mathrm{trio}\left(j\right)\right)}$$

Cov represents the covariance between the rescaled modes of two given trios. Var represents the variance of rescaled modes for each trio.

Finally, rescaled *K*_S_ ages of all focal-sister species pairs are plotted against the paralogous *K*_S_ distribution of the focal species, which can be further subjected to additional (log-scale) Gaussian Mixture Modeling (GMM) analysis, as implemented in wgd v1 (Zwaenepoel and Van de Peer 2018) and/or exponential-lognormal mixture modeling (ELMM) analysis, as implemented in ksrates (Sensalari *et al.* 2021), but in both node-averaged and node-weighted manners. The log-scale GMM analysis for an anchor pair *K*_S_ distribution uses the log-transformed *K*_S_ data to fit the Gaussian mixture model to delineate the potential WGD components, while the ELMM analysis fits the log-transformed whole-paranome *K*_S_ data to a mixture model consisting of an exponential component to model the background *K*_S_ distribution and up to five normal components to model the WGD events according to the expectation–maximization (EM) algorithm (Sensalari *et al.* 2021). An additional *K*_S_ tree method using orthogroups and user-defined tree topologies to examine the substitution rate variation was implemented in the program “wgd ksd.”

### 2.5 Absolute dating of WGDs

Although molecular clock analysis is a common practice to estimate the absolute age of gene duplication events, there has been no easy or straightforward method for estimating the age of WGD events. In what is probably the most significant advance of wgd v2, we implemented an integrated pipeline for WGD dating. The procedure is as follows. First, the anchor pair *K*_S_ distribution is inferred as described above. Second, only those anchor pairs residing within the 95% confidence level of the log-normal distribution fitted to each detected WGD peak are retained (using “wgd peak”). Additional manual filtering can be achieved by using the option “—kstodate” and a *K*_S_ saturation value typically 2 or 3 (Vanneste *et al.* 2012) can be set to filter anchor pairs by using the option “—kscutoff.” Third, the user has to provide a starting tree with a few other species and fossil calibration information. Fourth, orthogroups are constructed consisting of anchor pairs of the focal species and their reciprocal best hits (RBHs) against other species listed in the given starting tree via the program “wgd dmd.” Finally, the constructed orthogroups and the starting tree are passed on to the program “wgd focus” for the eventual dating of WGDs. Three alternative molecular dating approaches are implemented under the hood, namely mcmctree (Yang 2007), beast (Drummond *et al.* 2012), and r8s (Sanderson 2003). The detailed settings for each approach (prior distributions, MCMC settings, optimization parameters, etc.) can be controlled and customized by the users. Some guidelines about the dating approaches can be found in the online documentation.

### 2.6 Other functions

Other functions, such as orthogroups inference, which is also newly implemented in wgd v2, are described in the online documentation.

## 3 Results

### 3.1 Improved accuracy of whole-paranome inference

**Figure 1.**
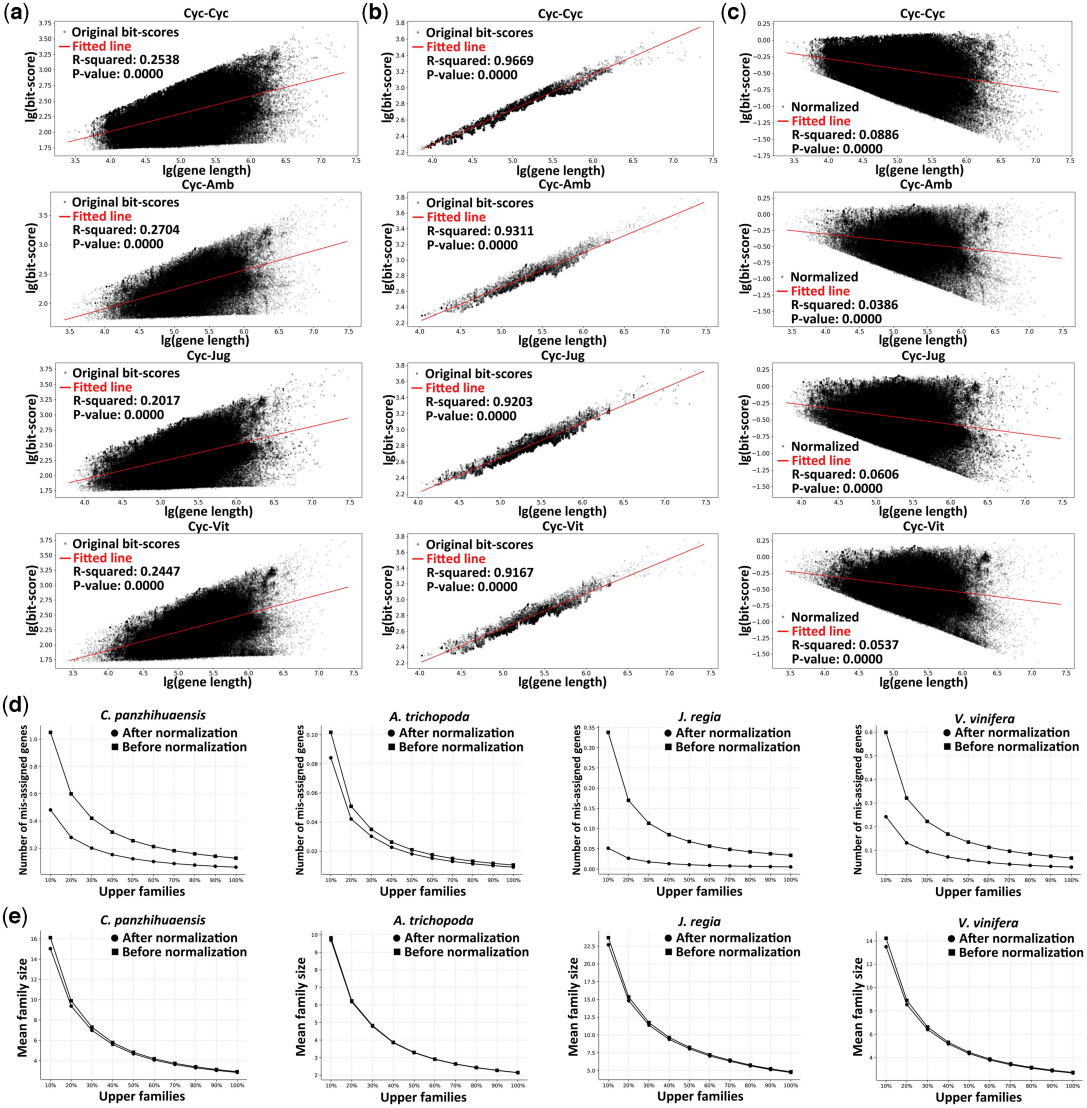
Gene length bias and the effect of normalization on whole-paranome inference. Cyc, Amb, Jug, and Vit represent *Cycas panzhihuaensis*, *Amborella trichopoda*, *Juglans regia*, and *Vitis vinifera*. Black dots show original or normalized bit-scores while red lines show fitted linear regressions. (a) Relationship between original bit-score and gene length of all hits for different species pairs. (b) Relationship between original bit-score and gene length of upper 5% hits per bin for different species pairs. (c) Relationship between normalized bit-score and gene length of all hits for different species pairs. (d) Number of mis-assigned genes, and (e) family size per paralogous gene family category in mean.

The intrinsic constraint that longer genes lead to larger bit-scores regardless of sequence similarities renders the bit-scores not suitable as a proxy for sequence similarity (Emms and Kelly 2019). A dataset consisting of four seed plant genomes, i.e. *Cycas panzhihuaensis*, *Amborella trichopoda*, *Juglans regia*, and *Vitis vinifera* was utilized to demonstrate this bias. As shown in Fig. 1a and b, before normalization, both intraspecific and interspecific hits of *C. panzhihuaensis* presented significant linear relationships between gene length and bit-score with *P*-values all smaller than 0.0001, and *R*-squared ranging from 0.20 to 0.27, while a clearer pattern was presented when only the upper 5% hits per bin were included, with *P*-values all smaller than 0.0001 and *R*-squared ranging from 0.92 to 0.97, indicating the intrinsic bias of gene length on bit-score. After normalization, the *R*-squared of overall hits declined to be all lower than 0.1, ranging from 0.04 to 0.09, suggesting a weak relationship at best between gene length and normalized bit-scores, as shown in Fig. 1c. For the inference of the whole-paranome, as shown in Fig. 1d and e and Supplementary Table S1, more gene families of smaller sizes were delineated after normalization, which exhibited higher accuracy in terms of consistently lower mean and overall number of mis-assigned genes per family category, particularly for *C. panzhihuaensis* and *V. vinifera*, where the *P*-values were smaller than 0.001 and 0.01 in both parametric and non-parametric tests.

### 3.2 Improved representation of intraspecific and interspecific collinearity

**Figure 2.**
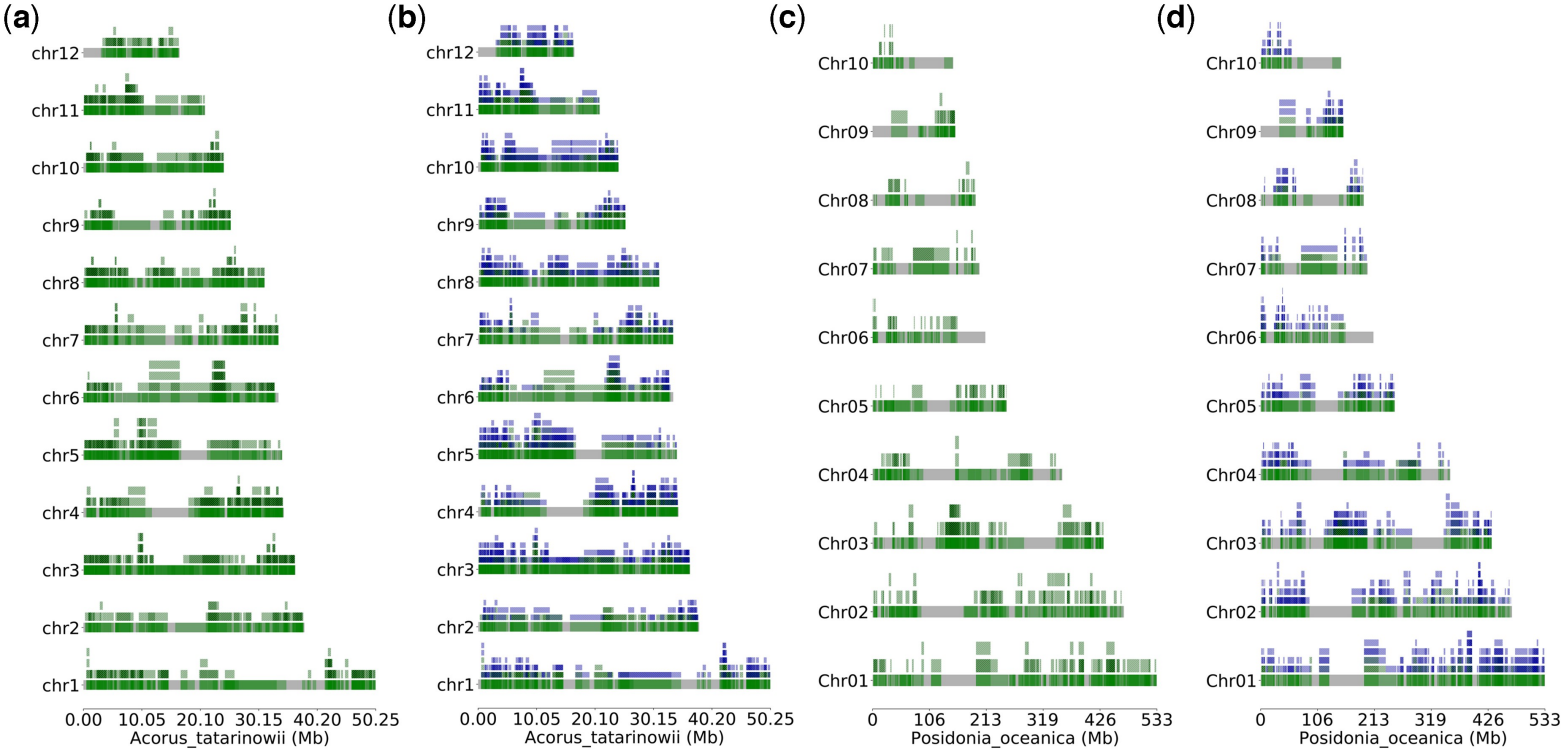
“dupStack” plots for *Acorus tatarinowii* and *Posidonia oceanica*. (a, c) show intraspecific multiplicon levels for *A. tatarinowii* and *P. oceanica*. (b, d) show interspecific multiplicon levels between *A. tatarinowii* and *P. oceanica* superimposed on the intraspecific multiplicon levels. Green “segments” denote intraspecific homology, while blue segments denote interspecific homology. The minimum length for segments is set to 100 kb. See text for details.

**Figure 3.**
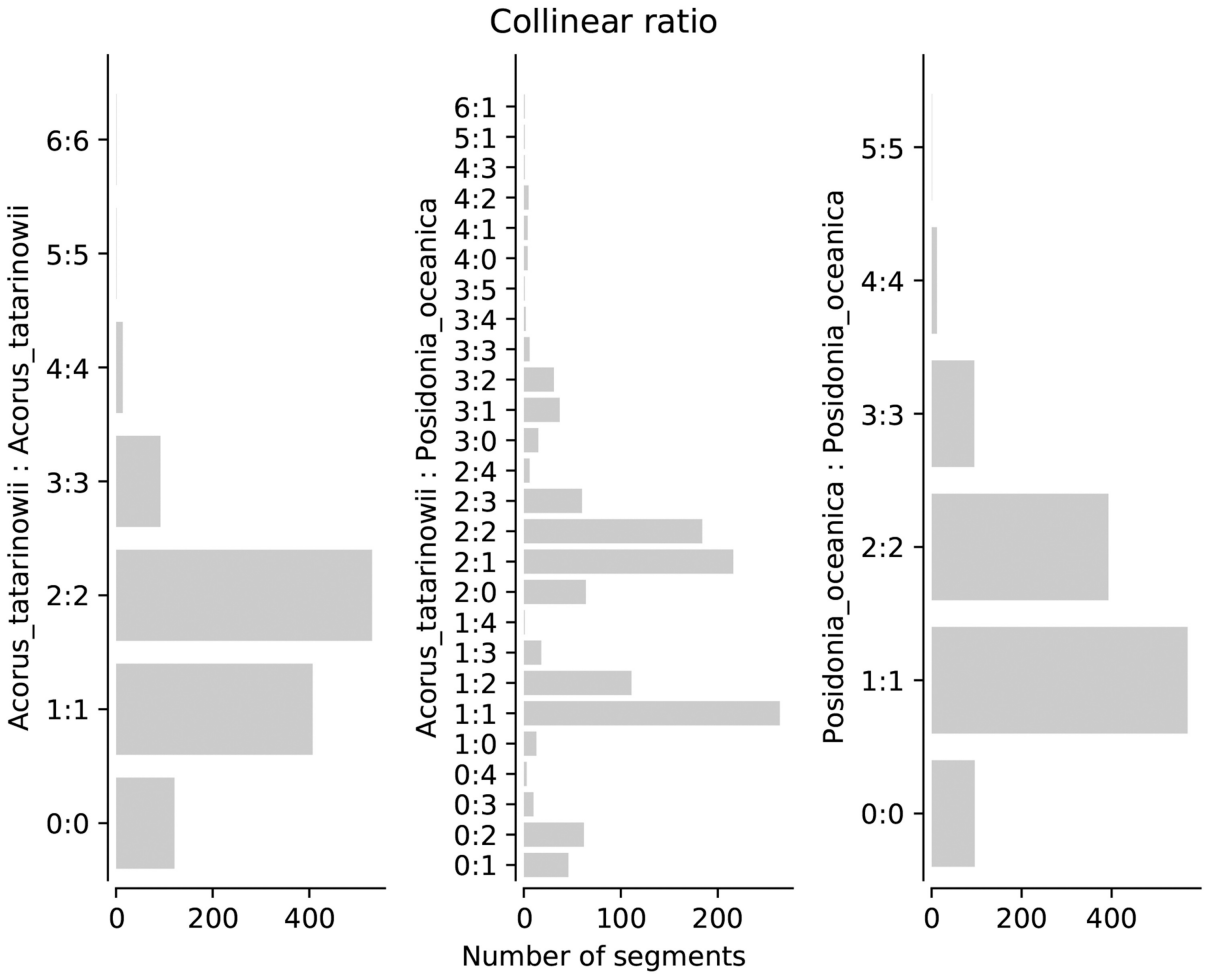
Intraspecific and interspecific homology collinearity levels for *Acorus tatarinowii* and *Posidonia oceanica*. The left and right panels show the intraspecific collinearity ratios of *A. tatarinowii* and *P. oceanica*, respectively. The middle panel shows the interspecific ratio of segmental collinearity. The minimum length for segments is set to 100 kb.

**Figure 4.**
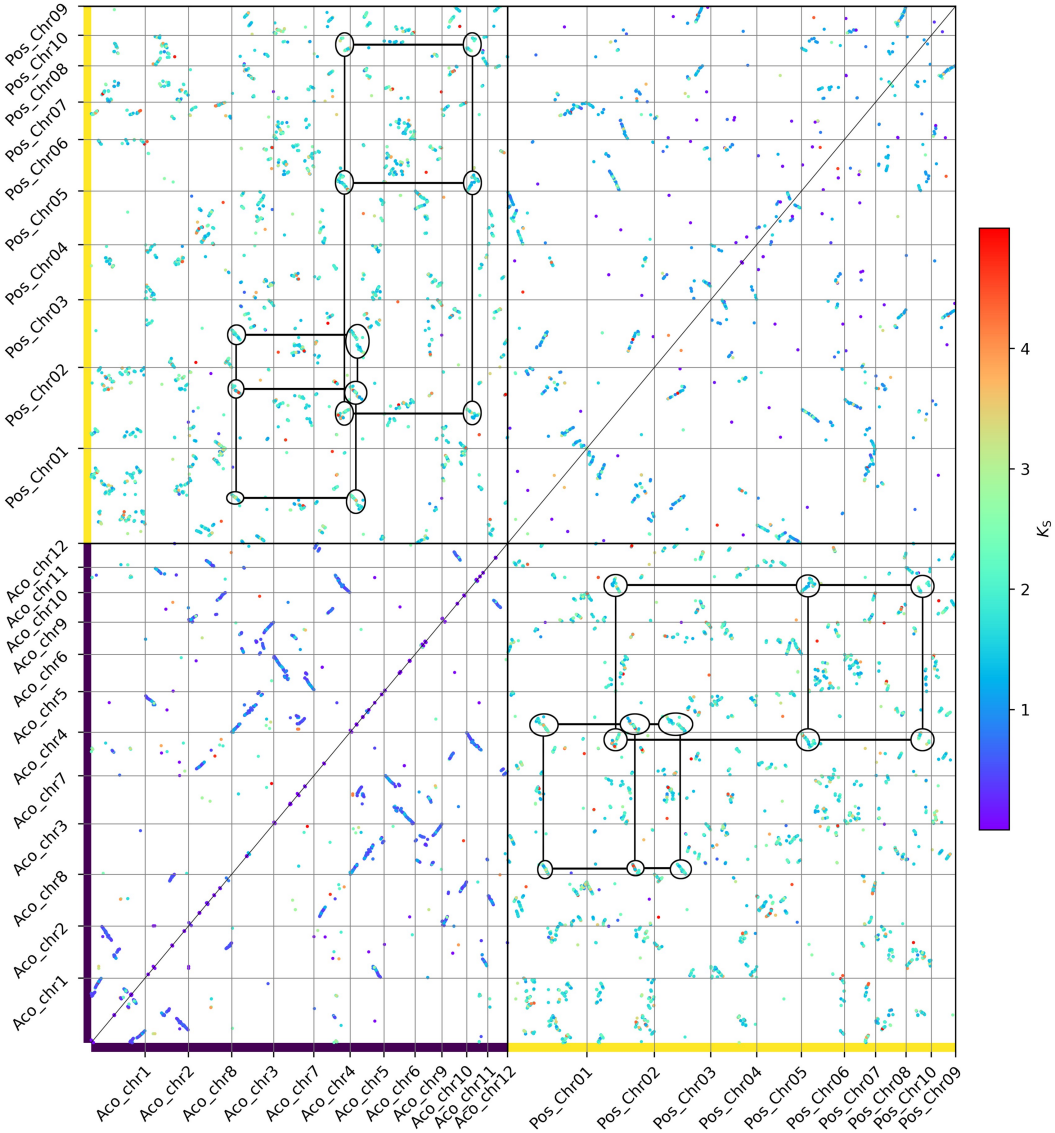
Intraspecific and interspecific genome homology dot plots for *Acorus tatarinowii* and *Posidonia oceanica*. Anchor pairs (homologous genes residing in homeologous segments) are represented as dots colored by their associated *K*_S_ value. Axes show genes on corresponding chromosomes. Marked circles denote collinear regions—with a ratio of 2:3—between *A. tatarinowii* and *P. oceanica.*

In wgd v2, we newly implemented the dupStack plots (Fig. 2) for the representation and visualization of multiplicons with different multiplication levels and Syndepth plots (Fig. 3) for the distribution of different categories of collinearity ratios within and between species, and improved the construction of gene homology matrix dot plots to show the collinearity between chrosomomes or contigs (Fig. 4). We chose the early diverging monocot species *Acorus tatarinowii* as an example, which was shown to have experienced a lineage-specific WGD after its divergence with other monocots (Shi *et al.* 2022) and compared this species with the seagrass *Posidonia oceanica*, which was shown to have experienced an ancient whole-genome triplication event shared by most, if not all, Alismatales (Ma *et al.* 2024). First, we constructed orthogroups for *A. tatarinowii* and *P. oceanica* using the built-in method in wgd v2 (see online documentation for details). Next, after the removal of redundant multiplicons and segments shorter than 100 kb, the chromosomes of *P. oceanica* and *A. tatarinowii* both harbored numerous duplicated and triplicated segments, while interspecific collinearity unveiled numerous triplicated segments in *P. oceanica* mapping to most of the chromosomes of *A. tatarinowii*, supporting the hexaploid nature of *P. oceanica* (Fig. 2) (Ma *et al.* 2024). Syndepth plots (Fig. 3) show the distribution of different levels of collinearity (number of homeologous segments) and again corroborated the large number of triplicated segments in both intraspecific and interspecific comparisons of *P. oceanica*. Remnants of the multiple rounds of WGDs experienced by *P. oceanica* and *A. tatarinowii* can be seen on most of the chromosomes (Fig. 4).

### 3.3 Correction of substitution rate and phylogenetic placement of WGDs

**Figure 5.**
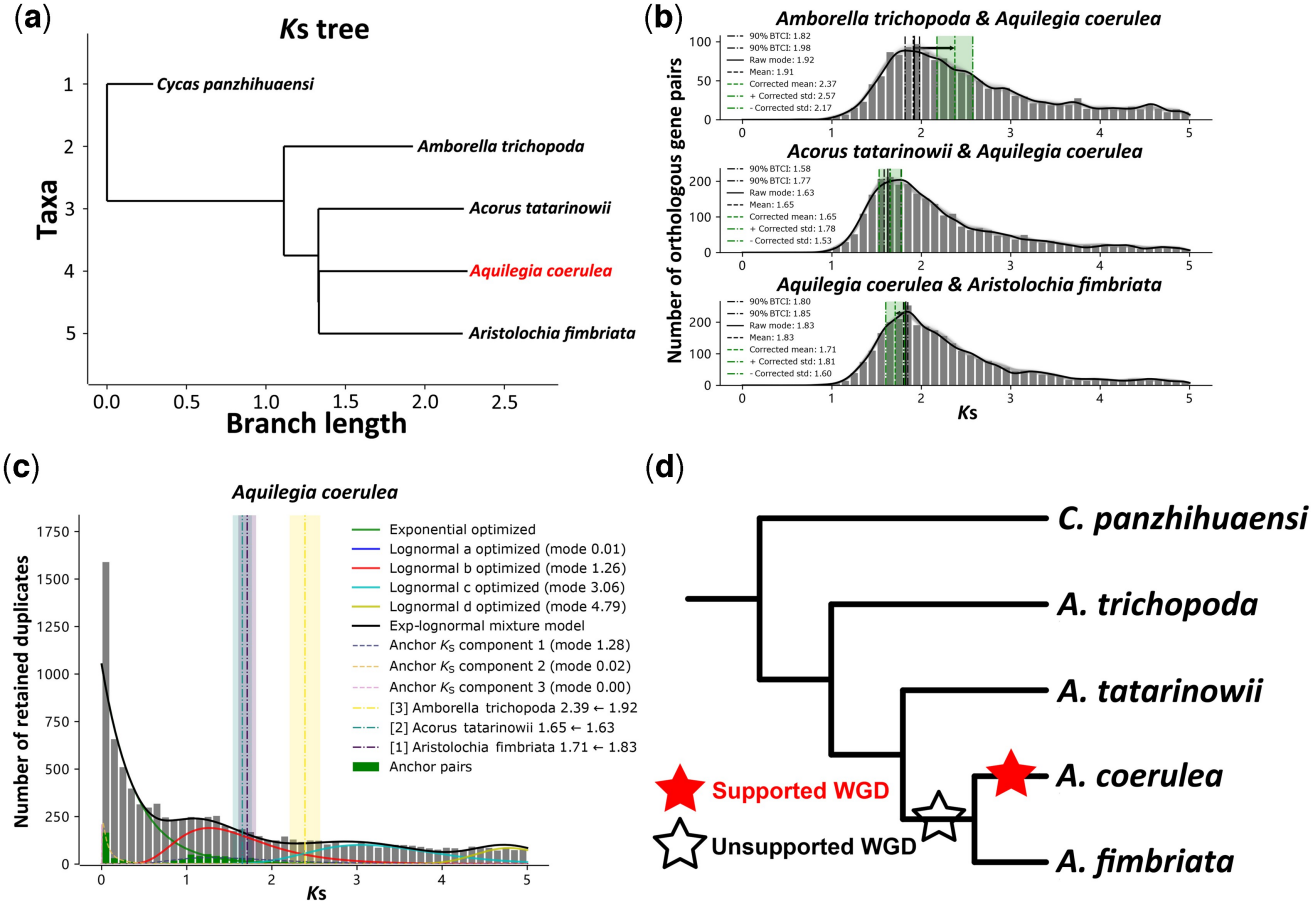
Synonymous substitution rate correction. (a) Inferred *K*_S_ tree showing the different synonymous substitution rates across branches (in particular between Amborella and the others) with focal species *Aquilegia coerulea* marked in red. (b) The orthologous *K*_S_ distributions between the focal species *A. coerulea* and three other sister species, with the mode of the fitted KDE on the “raw” *K*_S_ distribution marked by the black solid line; the mean and the 90% confidence interval of the mode from 200 bootstrap replicates marked by the black dashed and dash-dotted lines (BTCI); the mean and mean ± std of the corrected mode marked by the green dashes and dash-dotted lines with the area between the mean − std and mean + std filled in green; the direction of rate correction marked as black arrow; the KDE curve of 200 bootstrap replicates plotted in gray, and the KDE curve of the “raw” *K*_S_ distribution plotted in black. (c) *K*_S_ age distribution for the paranome of *A. coerulea*, with the inferred components of mixture modeling analysis of the whole-paranome and anchor pair *K*_S_ distribution plotted as solid and dashed lines and the corrected divergence times marked by the dash-dotted lines with the associated standard deviation. (d) The proposed scenario of the phylogenetic location of the WGD event in the *A. coerulea* lineage based on the results of mixture modeling and rate correction analysis (see text for details) illustrated in the cladogram.

The phylogenetic location of WGD events can be revealed by comparing the age of the WGD to divergence or speciation events. The “*K*_S_ age” of the same (shared) WGD or divergence event should be consistent across lineages assuming equal substitution rates. However, variation of substitution rates is found widespread in plant lineages (Mower *et al.* 2007, De La Torre *et al.* 2017), rendering the *K*_S_ a biased proxy of age. Only when the “*K*_S_ age” of WGD and divergence events is rescaled, based on a correction for different rates of synonymous substitutions, as shown in Fig. 5, we can reliably infer the phylogenetic placement of WGD events.

Here, we used the orthogroups consisting of RBHs across all species pairs of *Cycas panzhihuaensi*, *Amborella trichopoda*, *Acorus tatarinowii*, *Aquilegia coerulea*, and *Aristolochia fimbriata* to build orthologous *K*_S_ distributions and conduct synonymous substitution rate correction and mixture modeling analysis as implemented in wgd v2 with *A. coerulea* as the focal species. The phylogenetic relationships among Mesangiospermae adopted in this study follows Zeng and his colleagues (Zeng *et al.* 2014). As shown in the *K*_S_ tree of Fig. 5a, *A. trichopoda* (belonging to the so-called ANA clade) has a slower pace of accumulating synonymous substitutions compared to species from the Mesangiospermae clade. Provided the focal species *A. coerulea*, the divergence time between *A. coerulea* and *A. trichopoda* was rescaled from a *K*_S_ peak value (representing the mode of the distribution) of 1.91 to a *K*_S_ peak value of 2.37 after rate correction in wgd v2 (Fig. 5b). Mixture modeling (Fig. 5c) showed that the lognormal component b of the whole-paranome and the lognormal component 1 of anchor pair *K*_S_ distributions at peak values (representing the mode of the component) 1.26 and 1.28, respectively, are younger than the divergence of *A. coerulea* with other species, suggesting an ancient polyploidization unique to *A. coerulea* (Fig. 5d).

### 3.4 Absolute dating of WGD events

**Figure 6.**
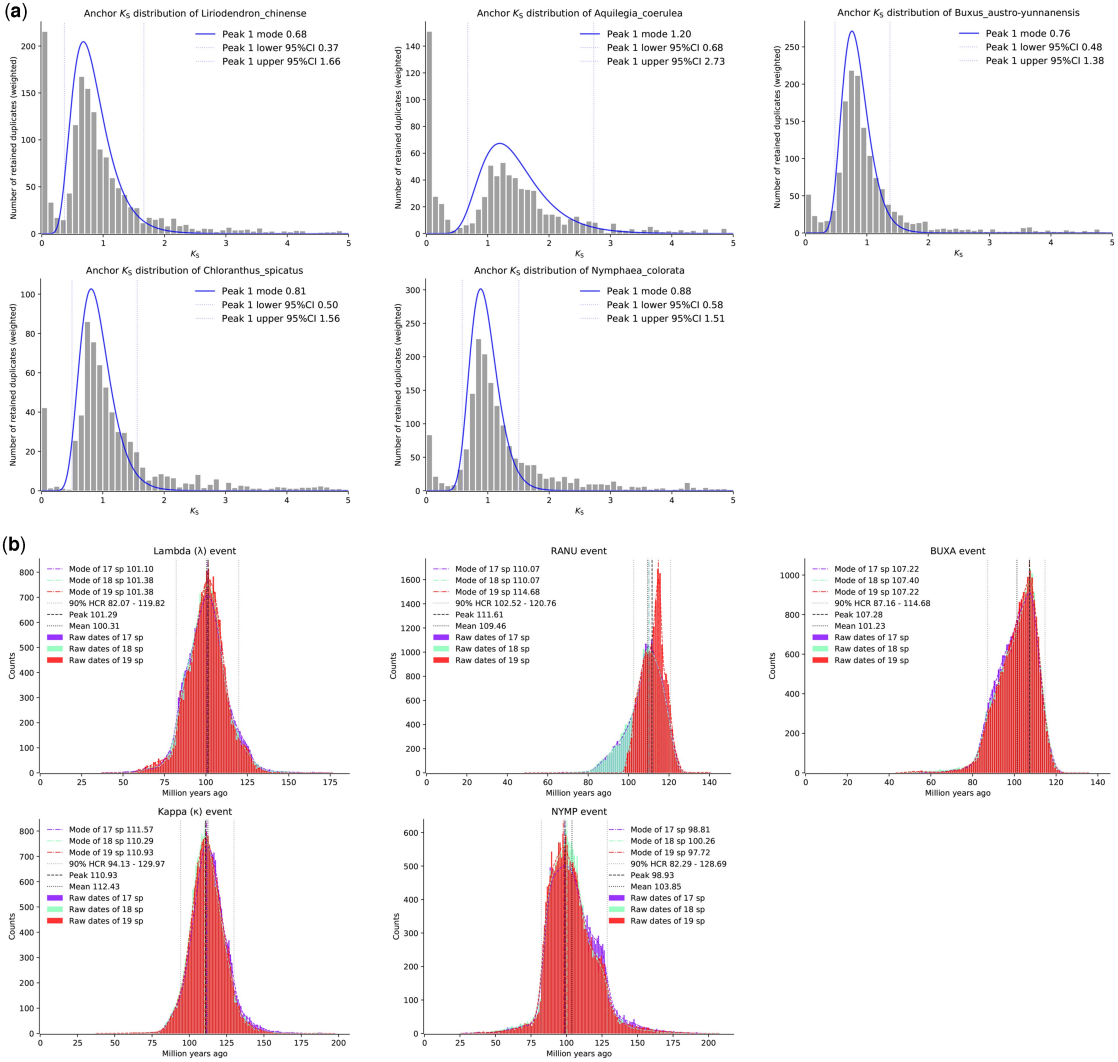
Anchor pair *K*_S_ and posterior date distributions of WGD dating for five angiosperms. (a) Anchor pair *K*_S_ distributions of five angiosperms with the associated 95% confidence level anchor pairs used for dating marked by the dotted lines, and the assumed log-normal distributions superimposed on the identified peaks. (b) Posterior distributions, KDE curves, modes, overall means, peaks and 90% HCR of the date estimation of the five WGD events (see text for details).

Determining the absolute age of a WGD can shed light on its evolutionary significance. As a demonstration of absolute dating of WGDs by wgd v2, we selected the following 5 species, *Liriodendron chinense*, *Aquilegia coerulea*, *Buxus austro-yunnanensis*, *Chloranthus spicatus*, and *Nymphaea colorata*, each of which has undergone ancient WGD events. We set the model parameters in mcmctree (v4.9j) as follows: we selected the independent rates model assuming a log-normal distribution of evolutionary rates across branches using an LG amino acid substitution matrix and assumed a gamma model with five rate categories and *α* = 0.5. Parameters controlling the birth-death process was set as 1 1 0.1 to generate uniform age priors on nodes that didn’t have a fossil calibration. Gamma priors for the transition/transversion rate ratio and shape parameter for variable rates among sites were set as 6 2 and 1 1. A Dirichlet-gamma prior was set upon the mean rate across loci and the variance in logarithm as 2 20 1 and 1 10 1. The first 2000 iterations were discarded as burn-in after which 20,000,000 iterations were performed with sampling per 1000 iterations. The species composition and adopted fossil calibrations of starting trees are shown in Supplementary Fig. S1 and Table S3. The effective sample size (ESS) of all parameters was found to be larger than 200, suggesting adequate sampling and convergence. To cope with species sampling bias, we perturbated by successively adding a single species and repeating the dating analysis to obtain a consensus WGD age from datasets with 17, 18 and 19 species, respectively. We adopted the 90% Highest Convergence Region (HCR) as the credible range of the inferred WGD age, calculated as the intersection of the 90% Highest Posterior Density (HPD) of date estimates from each dataset, while the averaged mode and overall mean were adopted as the reported peak and mean. We find that the 90% HCR, peak and mean of the WGD associated with the *L. chinense* lineage, marked as the lambda (*λ*) event following (Guo *et al.* 2021), were 82.07–119.82, 101.29, and 100.31 mya. Dating of the WGD in *A. coerulea*, marked as the RANU event, gave 102.52–120.76, 111.61, and 109.46 mya. Dating of the WGD in *B. austro-yunnanensis*, marked as the BUXA event, gave 87.16–114.68, 107.28, and 101.23 mya. Dating of the WGD in *C. spicatus*, marked as the kappa (*κ*) event following (Guo *et al.* 2021) gave 94.13–129.97, 110.93, and 112.43 mya. Finally, dating of the WGD in *N. colorata*, marked as the NYMP event, gave 82.29–128.69, 98.93, and 103.85 mya (Fig. 6).

## 4 Discussion

In this study, we present a major update of the earlier widely adopted wgd program (Zwaenepoel and Van de Peer 2018) for the inference and timing of ancient polyploidy events or WGDs. As illustrated with different plant genomes, the correction of gene length bias permits the construction of a more accurate paranome (the entire collection of duplicates in a genome). The improved representation of intraspecies and interspecies collinearity shed lights on the genomic landscape of species that have undergone different rounds of WGDs. The correction of substitution rate variation facilitates the correct phylogenetic placement of putative WGD events and absolute dating of ancient polyploidy or WGDs enables correlating duplication events with decisive moments in evolution. In conclusion, wgd v2 is a suite of tools greatly facilitating the inference of WGDs, and their relative and absolute timing.

## Supplementary Material

btae272_Supplementary_Data

## Data Availability

The genome assemblies involved in this paper were summarized in Supplementary Table S2.

## Code availability

The wgd v2 program can be found at https://github.com/heche-psb/wgd.
