# Supplementary material for: wgd v2: a suite of tools to uncover and date ancient polyploidy and whole-genome duplication
Source: Bioinformatics. 2024 Apr 17;40(5):btae272. doi: 10.1093/bioinformatics/btae272 (PMC11078771; doi:10.1093/bioinformatics/btae272)
Supplement: btae272_Supplementary_Data [file btae272_supplementary_data.zip › wgd_v2_Supplementary_Materials.docx]

**wgd v2: a suite of tools to uncover and date ancient polyploidy and whole-genome duplication**

Hengchi Chen^1,2†^, Arthur Zwaenepoel^3^, Yves Van de Peer^1,2,4,5†^

1. Department of Plant Biotechnology and Bioinformatics, Ghent University, 9052 Ghent, Belgium.
2. VIB Center for Plant Systems Biology, VIB, 9052 Ghent, Belgium.
3. UMR 8198, Evo-Eco-Paleo, University of Lille, CNRS, F-59000, Lille, France.
4. Centre for Microbial Ecology and Genomics, Department of Biochemistry, Genetics and Microbiology, University of Pretoria, Pretoria 0028, South Africa.
5. College of Horticulture, Academy for Advanced Interdisciplinary Studies, Nanjing Agricultural University, Nanjing, China.

^†^ Correspondence should be addressed to H.C. ([hengchi.chen@psb.vib-ugent.be](mailto:hengchi.chen@psb.vib-ugent.be)) and/or Y.V.d.P. ([yves.vandepeer@psb.vib-ugent.be](mailto:yves.vandepeer@psb.vib-ugent.be))

**Figure S1 | Fossil calibrations and starting tree topologies in the absolute dating of WGDs of five angiosperms.** a), b), c), d) and e) represent the starting tree topologies of *Liriodendron chinense*, *Aquilegia coerulea*, *Buxus austro-yunnanensis*, *Chloranthus spicatus* and *Nymphaea colorata*, respectively. The left, middle and right panels denote the 17, 18 and 19 species datasets, respectively. Fossil calibration points are denoted on the corresponding nodes with lower and higher bounds (in 100 million years).

**Table S1 | T-test and Mann–Whitney U test of mis-assigned genes for each gene family category.** P-values and T-statistic are on the left and right side of the comma in the column T-test. P-values and Mann-Whitney U-statistic are given on the left and right side of the comma in the column Mann–Whitney U test, respectively. P-values smaller than 1e-04 are all denoted as <1e-04. The number of mis-assigned genes after and before normalization is on the left and right side of the slash in the column Overall number, respectively.

| **Species** | **Upper family** | **T-test** | **Mann–Whitney U test** | **Overall number** |
| --- | --- | --- | --- | --- |
| ***Cycas panzhihuaensis*** | 10% | <1e-04, -4.196 | 2.412e-04, 7.666e+04 | 209/421 |
|  | 20% | <1e-04, -4.487 | <1e-04, 3.203e+05 | 243/480 |
|  | 30% | <1e-04, -4.527 | <1e-04, 7.370e+05 | 262/505 |
|  | 40% | <1e-04, -4.507 | 1.295e-04, 1.329e+06 | 267/511 |
|  | 50% | <1e-04, -4.483 | 1.820e-04, 2.096e+06 | 268/512 |
|  | 60% | <1e-04, -4.467 | 2.243e-04, 3.036e+06 | 268/512 |
|  | 70% | <1e-04, -4.456 | 2.595e-04, 4.149e+06 | 268/512 |
|  | 80% | <1e-04, -4.447 | 2.889e-04, 5.434e+06 | 268/512 |
|  | 90% | <1e-04, -4.441 | 3.137e-04, 6.893e+06 | 268/512 |
|  | 100% | <1e-04, -4.435 | 3.352e-04, 8.530e+06 | 268/512 |
| ***Amborella trichopoda*** | 10% | 0.633, -0.478 | 0.464, 4.65e+04 | 26/31 |
|  | 20% | 0.639, -0.470 | 0.475, 1.869e+05 | 26/31 |
|  | 30% | 0.701, -0.384 | 0.588, 4.217e+05 | 27/31 |
|  | 40% | 0.705, -0.379 | 0.599, 7.501e+05 | 28/32 |
|  | 50% | 0.705, -0.379 | 0.600, 1.17e+06 | 28/32 |
|  | 60% | 0.705, -0.379 | 0.600, 1.689e+06 | 28/32 |
|  | 70% | 0.705, -0.378 | 0.602, 2.299e+06 | 28/32 |
|  | 80% | 0.704, -0.379 | 0.601, 3.003e+06 | 28/32 |
|  | 90% | 0.705, -0.378 | 0.602, 3.801e+06 | 28/32 |
|  | 100% | 0.705, -0.378 | 0.602, 4.695e+06 | 28/32 |
| ***Juglans regia*** | 10% | 0.161, -1.401 | 0.148, 1.737e+05 | 31/198 |
|  | 20% | 0.162, -1.400 | 0.164, 7.009e+05 | 32/199 |
|  | 30% | 0.161, -1.401 | 0.166, 1.580e+06 | 32/199 |
|  | 40% | 0.162, -1.400 | 0.168, 2.813e+06 | 32/199 |
|  | 50% | 0.162, -1.400 | 0.169, 4.398e+06 | 32/199 |
|  | 60% | 0.162, -1.400 | 0.170, 6.338e+06 | 32/199 |
|  | 70% | 0.162, -1.400 | 0.170, 8.627e+06 | 32/199 |
|  | 80% | 0.162, -1.400 | 0.170, 1.127e+07 | 32/199 |
|  | 90% | 0.162, -1.400 | 0.171, 1.427e+07 | 32/199 |
|  | 100% | 0.162, -1.400 | 0.171, 1.762e+07 | 32/199 |
| ***Vitis vinifera*** | 10% | 2.282e-03, -3.061 | 5.954e-03, 7.602e+04 | 101/236 |
|  | 20% | 1.545e-03, -3.172 | 4.430e-03, 3.144e+05 | 110/253 |
|  | 30% | 1.427e-03, -3.193 | 5.637e-03, 7.173e+05 | 118/263 |
|  | 40% | 1.476e-03, -3.182 | 6.989e-03, 1.283e+06 | 121/266 |
|  | 50% | 1.618e-03, -3.155 | 9.537e-03, 2.016e+06 | 122/266 |
|  | 60% | 1.531e-03, -3.171 | 8.242e-03, 2.911e+06 | 122/267 |
|  | 70% | 1.546e-03, -3.167 | 8.570e-03, 3.969e+06 | 122/267 |
|  | 80% | 1.558e-03, -3.165 | 8.826e-03, 5.193e+06 | 122/267 |
|  | 90% | 1.568e-03, -3.163 | 9.029e-03, 6.581e+06 | 122/267 |
|  | 100% | 1.575e-03, -3.162 | 9.193e-03, 8.134e+06 | 122/267 |

**Table S2 | Taxonomy and data source of species involved in this study.**

| Clade | Order | Family | Species | Source of data |
| --- | --- | --- | --- | --- |
| Acrogymnospermae | Cycadales | Cycadaceae | *Cycas panzhihuaensis* | [CNGB](https://db.cngb.org/codeplot/datasets/public_dataset?id=PwRftGHfPs5qG3gE) |
| Monocotyledoneae | Acorales | Acoraceae | *Acorus tatarinowii* | [CNGB](https://ftp.cngb.org/pub/CNSA/data4/CNP0001708/CNS0456199/CNA0036157/) |
| Monocotyledoneae | Acorales | Acoraceae | *Acorus americanus* | Phytozome V13 |
| Monocotyledoneae | Alismatales | Posidoniaceae | *Posidonia oceanica* | [BEG](https://bioinformatics.psb.ugent.be/gdb/seagrasses/Posidonia_oceanica/) |
| Monocotyledoneae | Alismatales | Araceae | *Spirodela intermedia* | [NCBI](https://www.ncbi.nlm.nih.gov/data-hub/genome/GCA_902729315.2/) |
| Monocotyledoneae | Alismatales | Araceae | *Amorphophallus konjac* | [Figshare](https://figshare.com/articles/online_resource/A_chromosome-level_genome_assembly_of_Amorphophallus_konjac_provides_insights_into_glucomannan_biosynthesis/15169578) |
| Monocotyledoneae | Poales | Poaceae | *Brachypodium hybridum* | Phytozome V13 |
| Monocotyledoneae | Poales | Cyperaceae | *Rhynchospora pubera* | [CoGe](https://genomevolution.org/CoGe/SearchResults.pl?s=Rhynchospora%20pubera&p=genome) |
| Monocotyledoneae | Arecales | Arecaceae | *Phoenix dactylifera* | [NCBI](https://www.ncbi.nlm.nih.gov/data-hub/genome/GCF_009389715.1/) |
| Monocotyledoneae | Arecales | Arecaceae | *Elaeis guineensis* | [NCBI](https://www.ncbi.nlm.nih.gov/data-hub/genome/GCF_000442705.1/) |
| Monocotyledoneae | Asparagales | Orchidaceae | *Dendrobium nobile* | [NCBI](https://www.ncbi.nlm.nih.gov/data-hub/genome/GCA_022539455.1/) |
| Monocotyledoneae | Asparagales | Orchidaceae | *Phalaenopsis equestris* | [NCBI](https://www.ncbi.nlm.nih.gov/data-hub/genome/GCF_001263595.1/) |
| Monocotyledoneae | Asparagales | Asparagaceae | *Asparagus setaceus* | [Dryad](https://datadryad.org/stash/dataset/doi:10.5061/dryad.1c59zw3rm) |
| Monocotyledoneae | Dioscoreales | Dioscoreaceae | *Dioscorea alata* | Phytozome V13 |
| Monocotyledoneae | Pandanales | Velloziaceae | *Acanthochlamys bracteata* | [NGDC](https://ngdc.cncb.ac.cn/search/?dbId=gwh&q=GWHBAYO00000000.1&page=1) |
| Magnoliids | Magnoliales | Magnoliaceae | *Liriodendron chinense* | [TreeGenes](https://treegenesdb.org/) |
| Magnoliids | Piperales | Saururaceae | *Saururus chinensis* | [NGDC](https://ngdc.cncb.ac.cn/search/?dbId=gwh&q=GWHBMBM00000000&page=1) |
| Magnoliids | Piperales | Aristolochiaceae | *Aristolochia fimbriata* | [NGDC](https://ngdc.cncb.ac.cn/search/?dbId=gwh&q=PRJCA004207&page=1) |
| Magnoliids | Piperales | Aristolochiaceae | *Aristolochia contorta* | [CoGe](https://genomevolution.org/coge/SearchResults.pl?s=Aristolochia%20contorta&p=genome) |
| Chloranthales | Chloranthales | Chloranthaceae | *Chloranthus spicatus* | [NGDC](https://ngdc.cncb.ac.cn/search/?dbId=gwh&q=GWHBFSJ00000000&page=1) |
| ANA clade | Nymphaeales | Nymphaeaceae | *Nymphaea colorata* | Phytozome V13 |
| ANA clade | Amborellales | Amborellaceae | *Amborella trichopoda* | Phytozome V13 |
| Eudicotyledoneae | Ranunculales | Ranunculaceae | *Aquilegia coerulea* | Phytozome V13 |
| Eudicotyledoneae | Ranunculales | Ranunculaceae | *Aquilegia oxysepala* | [PLAZA](https://bioinformatics.psb.ugent.be/plaza/versions/plaza_v5_dicots/organism/view/Aquilegia+oxysepala) |
| Eudicotyledoneae | Buxales | Buxaceae | *Buxus austro−yunnanensis* | [NGDC](https://ngdc.cncb.ac.cn/gwh/Assembly/24404/show) |
| Eudicotyledoneae | Proteales | Nelumbonaceae | *Nelumbo nucifera* | [NCBI](https://www.ncbi.nlm.nih.gov/data-hub/genome/GCF_000365185.1/) |
| Eudicotyledoneae | Proteales | Proteaceae | *Protea cynaroides* | [NCBI](https://www.ncbi.nlm.nih.gov/data-hub/genome/GCA_028583415.1/) |
| Eudicotyledoneae | Proteales | Proteaceae | *Macadamia integrifolia* | [Southern Cross University data repository](https://researchportal.scu.edu.au/esploro/outputs/dataset/991012821204402368) |
| Eudicotyledoneae | Vitales | Vitaceae | *Vitis arizonica* | [Zenodo](https://zenodo.org/record/4977234#.Y3oq4HbMKUc) |
| Eudicotyledoneae | Vitales | Vitaceae | *Vitis vinifera* | Phytozome V13 |
| Eudicotyledoneae | Caryophyllales | Amaranthaceae | *Beta vulgaris* | Phytozome V13 |
| Eudicotyledoneae | Caryophyllales | Simmondsiaceae | *Simmondsia chinensis* | [NGDC](https://ngdc.cncb.ac.cn/search/?dbId=gwh&q=GWHAASQ00000000+) |
| Eudicotyledoneae | Ericales | Ericaceae | *Vaccinium corymbosum* | [CNGB](https://ftp.cngb.org/pub/gigadb/pub/10.5524/100001_101000/100537/) |
| Eudicotyledoneae | Aquifoliales | Aquifoliaceae | *Ilex polyneura* | [NGDC](https://ngdc.cncb.ac.cn/search/?dbId=gwh&q=GWHBDNW00000000) |
| Eudicotyledoneae | Gentianales | Rubiaceae | *Chiococca alba* | [Dryad](https://datadryad.org/stash/dataset/doi:10.5061/dryad.00000000r) |

**Table S3 | Fossil calibrations involved in this study following (Morris et al. 2018) and (Barba-Montoya et al. 2018).**

| Clade | Minimum age/mya | Maximum age/mya |
| --- | --- | --- |
| Crown Mesangiospermae | 125.9 | 247.2 |
| Crown Monocotyledoneae | 113 | 128.63 |
| Unnamed* | 85.8 | 128.63 |
| Crown Arecales | 83.41 | 128.63 |
| Crown Magnoliids | 110.87 | 247.2 |
| Crown Piperales | 44.3 | 247.2 |
| Unnamed2** | 85.8 | 128.63 |
| Crown Eudicotyledoneae | 119.6 | 128.63 |
| Crown Proteales | 107.59 | 128.63 |
| Crown Angiospermae | 125.9 | 247.2 |

* Crown group of [[Dioscoreales + Pandanales] + [Asparagales + [Aracales + Poales]]].

** Crown group of [Superrosids + Superasterids].

**References**

Barba-Montoya J, dos Reis M, Schneider H et al. Constraining uncertainty in the timescale of angiosperm evolution and the veracity of a Cretaceous Terrestrial Revolution. *New Phytologist* 2018;**218**:819-834.

Morris JL, Puttick MN, Clark JW et al. The timescale of early land plant evolution. *Proceedings of the National Academy of Sciences* 2018;**115**:E2274-E2283.
